# MATTERS OF THE HEART: DAILY SOCIAL INTERACTIONS AND CARDIOVASCULAR REACTIVITY IN MIDDLE AND OLD AGE

**DOI:** 10.1093/geroni/igz038.2716

**Published:** 2019-11-08

**Authors:** Kira S Birditt, Angela Turkelson, Meaghan Mones, Kayvan Najarian, Richard Gonzalez

**Affiliations:** 1 Institute for Social Research, University of Michigan, Ann Arbor, Michigan, United States; 2 Computational Medicine, University of michigan, ann Arbor, Michigan, United States

## Abstract

Social ties are essential for survival but the mechanisms accounting for this link are unclear. This study examined links between daily interpersonal experiences and cardiovascular reactivity. A total of 34 participants (aged 40 to 80) completed ecological momentary assessment surveys every three hours for 4 days and wore a device that assessed heart rate (HR) and heart rate variability (HRV). Multilevel models revealed that a greater number of social interactions and negative social interactions predicted increased HR. Links between social interactions and cardiovascular reactivity varied by gender and race. A greater number of interactions and negative interactions predicted increased HRV among men and not women. A greater number of social interactions predicted increased HR among Black individuals and White women but not White men. Thus, social interactions appear to get under the skin via the cardiovascular system but in unique ways that vary by gender and race.

